# A Comparative Analysis of Bupivacaine Concentrations in Adductor Canal Block for Pain Management Post-unilateral Knee Arthroplasty

**DOI:** 10.5812/aapm-161215

**Published:** 2025-07-23

**Authors:** Hosnieh Esmaeili Nameghi, Amir Shahriar Ariamanesh, Mehrdad Mokarram Dori, Vahideh Ghorani, Malihe Aghasizadeh, Seyed-Hossein Khademi

**Affiliations:** 1 Department of Anesthesiology, School of Medicine, Mashhad University of Medical Sciences, Mashhad, Iran; 2 Department of Orthopedics, School of Medicine, Mashhad University of Medical Sciences, Mashhad, Iran; 3 Clinical Research Development Unit, Imam Reza Hospital, Faculty of Medicine, Mashhad University of Medical Sciences, Mashhad, Iran; 4 Applied Biomedical Research Center, Mashhad University of Medical Sciences, Mashhad, Iran; 5 Vascular and Endovascular Surgery Research Center, Mashhad University of Medical Sciences, Mashhad, Iran

**Keywords:** Postoperative, Pain, Arthroplasty, Bupivacaine

## Abstract

**Background:**

The adductor canal block (ACB) is a widely recognized intervention for post-surgical pain management following total knee arthroplasty (TKA).

**Objectives:**

In this study, we evaluated the analgesic efficacy and functional outcomes of ACB between bupivacaine at concentrations of 0.5% and 0.25% in participants who underwent primary unilateral TKA.

**Methods:**

In this randomized controlled trial, we monitored participants who had undergone TKA surgery. They were randomly assigned to receive postoperative ACB with either 0.5% bupivacaine (22 patients) or 0.25% bupivacaine (22 patients). Data were collected at various time points following the intervention, including quadriceps muscle tone assessed by the Manual Muscle Contraction Test (MMT), pain levels measured using the Visual Analog Scale (VAS) pain scores, analgesic consumption, and patient satisfaction with pain control.

**Results:**

There was no significant difference in pain intensity between the two groups three hours after surgery (P = 0.55). However, the group receiving 0.5% bupivacaine showed a statistically significant trend toward lower VAS scores at 6, 12, and 24 hours post-operation compared to the 0.25% bupivacaine group (P = 0.02, P < 0.005, and P = 0.002, respectively). Patients' satisfaction with postoperative pain management and quadriceps muscle strength did not differ significantly between the two groups. Similarly, opioid consumption at 3, 6, and 24 hours post-surgery showed no significant difference (P = 0.052, P = 0.43). However, opioid consumption was notably higher in the 0.25% bupivacaine group 12 hours after surgery compared to the 0.5% bupivacaine group (P = 0.002).

**Conclusions:**

This study demonstrates that a higher dose of bupivacaine plays a crucial role in effectively reducing postoperative pain and minimizing the need for narcotic consumption.

## 1. Background

Peripheral femoral nerve blockade (FNB) is a widely used technique for managing perioperative pain in patients undergoing total knee arthroplasty (TKA), especially those with advanced rheumatoid arthritis or osteoarthritis. It is estimated that the number of TKA procedures will reach 3.5 million by 2030 (1, 2). Postoperative pain following TKA can hinder early mobilization and range of motion, increasing the risk of thromboembolism, and impacts rehabilitation, patient satisfaction, and overall outcomes (3, 4). Postoperative pain is associated with reduced knee mobility, extended hospital stays, and a high risk of complications. Despite the implementation of comprehensive multimodal analgesic regimens, this issue has not been entirely resolved (5, 6). However, the widespread use of local anesthesia following knee joint replacement surgery has led to improved pain management, accelerated functional recovery, and shorter hospital stays (7).

Regional anesthesia techniques, such as FNBs and adductor canal blocks (ACBs), are commonly used for pain management in patients undergoing TKA. While FNB is widely employed in TKA, it can lead to quadriceps muscle weakness, often necessitating the use of a knee immobilizer after surgery, which may hinder early ambulation and extend hospitalization (8-10). Additionally, FNBs typically require a continuous infusion pump. In contrast, ACB is a relatively newer peripheral nerve blockade technique introduced by Lund et al. (11). This technique offers better patient control after TKA compared to FNB. It primarily affects sensory nerve blocking rather than motor nerve blocking, offering the advantage of potentially preserving muscle strength. The ACB targets two major sensory nerves from the femoral nerve (FN) to the knee — the vastus medialis branch and the saphenous nerve — as well as the articular parts of the obturator nerve. As a result, it largely maintains quadriceps muscle strength (12, 13). However, no definitive clinical studies have established the optimal volume or dose of local anesthetic for ACB in postoperative pain management for TKA. It appears that dose-volume manipulation plays a crucial role in the effectiveness of the ACB for post-surgery pain relief. The choice of 0.25% and 0.5% bupivacaine concentrations is based on standard clinical practice and prior research. Typically, these concentrations are compared to evaluate the balance between analgesic efficacy and side effects such as motor blockade. A lower concentration (0.25%) may be expected to provide adequate pain relief with minimal motor impairment, while a higher concentration (0.5%) could potentially offer stronger analgesia but with an increased risk of affecting muscle function.

## 2. Objectives

This research was conducted to compare the effect of ACB with bupivacaine 0.5% and 0.25% in cases undergoing unilateral total knee replacement surgery.

## 3. Methods

This study was conducted as a randomized double-blind controlled trial and received approval from the Mashhad University of Medical Sciences (ethics approval code: IR.MUMS.MEDICAL.REC.1400.802; clinical trial code: IRCT20220731055587N1). The study sample included 44 patients scheduled for unilateral elective knee arthroplasty at Imam Reza Hospital in Mashhad, Iran, between September 2022 and March 2023.

Inclusion criteria included patients requiring unilateral knee arthroplasty classified as American Society of Anesthesiology (ASA) class 1 and 2, age over 18 years, Body Mass Index (BMI) below 35, and without a history of neuropathy, coagulopathy, and drug addiction. Exclusion criteria included sensitivity to local anesthetic drugs, infection at the injection site, inability to measure pain, history of elective arthroscopic knee surgery, bilateral arthroplasty, lack of consent to participate in the study, contraindications for regional anesthesia, chronic musculoskeletal pain, and psychiatric medication use.

The participants were divided into two groups by block randomization in a double-blind manner (both patient and assessor blinded). They were supposed to be uninformed. The individuals performing the block were not involved in data collection and evaluation. As premedication, the patients were given 1 g of acetaminophen and 400 mg of ibuprofen an hour before the operation. Additionally, they were administered 8 mg of dexamethasone intravenously to prevent postoperative nausea. Spinal anesthesia was administered with 25 μg of fentanyl and 2 to 2.5 mL of 0.5% hyperbaric bupivacaine in the space between L3 - L4 (or alternatively L2 - L3 or L4 - L5). Sedation with fluid therapy and propofol was applied during the operation. A femoral tourniquet was applied to all patients before the operation.

After the surgery and before dressing the knee under sterile conditions, while the patient was lying on their back and the knee was slightly rotated outward (to expose the inside of the thigh), the ACB was performed. A linear and high-frequency (6 - 15 MHz) ultrasound probe, sheathed in a sterile cover, was positioned transversely to visualize the adductor canal in the anteromedial part of the thigh at the junction between the middle and distal thirds of the thigh as a short axis. The vastus medialis muscle (external), sartorius muscle (anterior), and femoral artery (internal) determined the borders of the canal. A 22-gauge, 5 cm short bevel needle was utilized in-plane with the transducer, from lateral to medial, with the needle tip targeted anterolateral to the femoral artery and below the sartorius. To perform the intervention in the control group, a volume of 10 mL of bupivacaine 0.5% was administered after a careful negative aspiration through the injection port of the needle. In the intervention group, a volume of 10 mL of bupivacaine 0.25% was injected in the same manner. The spread of the drug between the sartorius and the femoral artery was observed in real-time on ultrasound. Then, the wound was bandaged, and the patient was taken to recovery.

After surgery, to equalize the state of pain control in both groups, 1 g of Apotel was infused every 6 hours, and 100 mg of diclofenac was administered rectally every 12 hours. In case of insufficient relief, a bolus of 5 mg morphine was injected, and the amount of additional analgesic was recorded and calculated. The Numeric Rating Scale was used to evaluate pain (1 - 10, with 1 being the least and 10 being the worst pain described by the participant) at 3, 6, 12, and 24 hours after the operation. The score of non-satisfaction was considered equal to zero, and full satisfaction was considered equal to 10. The strength of the quadriceps muscle was evaluated by the manual muscle contraction test (MMT) on a 0 - 5 scale. Additionally, the amount of analgesic consumption and the level of patient satisfaction with pain control were evaluated and compared between the two groups.

### 3.1. Statistical Methods and Sample Size

The sample size was calculated by considering the average difference of 2 pain scores between the two groups (by default based on previous observations). Considering an alpha error of 50% and the power of the test at 80%, the average pain in the group receiving ACB with bupivacaine 0.25% was calculated as (2.6) 3.8 based on the Memtsoudis et al. study, with a sample size of 22 participants in each group (14). Finally, considering a 10% loss, the final sample volume was calculated to be 24 people in each group.

SPSS version 27 software was utilized for data analysis. First, demographic data and general characteristics of patients were documented using descriptive statistics methods, including central indices, dispersion indices, and frequency distribution. Next, the correlation between different qualitative variables was measured using the chi-square and Fisher's exact statistical tests. The normality or abnormality of the distribution of quantitative data was also checked by utilizing the Kolmogorov-Smirnov test. If the distribution was normal, the independent *t*-test was utilized to compare the quantitative variables between the two groups. Mann-Whitney's statistical test was used in cases where the distribution was not normal. In all statistical analyses, P < 0.05 was considered a significant level.

## 4. Results

Forty-four candidates for unilateral TKA were enrolled, with 22 patients in each group (Table 1). The findings indicate no significant differences in age, height, weight, or BMI between the groups.

**Table 1.** Baseline Characteristics of Patients ^a^

Variables	Bupivacaine 0.25%	Bupivacaine 0.5%	P-Value
**Age (y)**	64.09 ± 9.83	56.85 ± 14.19	0.058
**Height (cm) **	160.95 ± 5.35	165.23 ± 8.28	0.007
**Weight (kg) **	70.04 ± 10	72.63 ± 12.74	0.45
**BMI (kg/m** ^**2**^ **)**	27.04 ± 3.51	26.46 ± 3.55	0.59

Abbreviation: BMI, Body Mass Index.

^a^ Values are expressed as mean ± SD.

No significant difference was documented in pain intensity 3 hours post-surgery between the two groups (P = 0.55, Z = 0.59). However, at 6 hours (P = 0.02, *t* = -2.3), 12 hours (P < 0.005, Z = -3.56), and 24 hours (P = 0.002, Z = -3.16) after the operation, the pain intensity was notably different between the two groups of patients. Patients' satisfaction with postoperative pain control was not notably different between the two groups. No notable difference was observed in the quadriceps muscle strength score 3 hours after the operation between the groups. Additionally, no difference was documented in the proportion of muscle strength at 6, 12, and 24 hours after surgery. Results are presented in Table 2. 

**Table 2.** Comparison of Main Variables at Different Times After Surgery in Two Groups ^a^

Variables	3 Hours	6 Hours	12 Hours	24 Hours
**Pain score**				
Bupivacaine 0.5%	2.95 ± 1.46	4.909 ± 1.77	4.18 ± 1.43	4.45 ± 1.65
Bupivacaine 0.25%	3.22 ± 1.77	6.09 ± 1.63	6 ± 1.66	6.09 ± 1.44
P-value	0.55	0.02	> 0.005	0.002
**Satisfactory from pain controlling**				
Bupivacaine 0.5%	9.18 ± 1	8.77 ± 1.02	8.63 ± 1.04	8.45 ± 1.33
Bupivacaine 0.25%	9 ± 1.15	8.45 ± 1.22	8.09 ± 1.57	8.31 ± 1.52
P-value	0.64	0.44	0.26	0.68
**Quadriceps strength scores**				
Bupivacaine 0.5%	1.04 ± 2.24	1.09 ± 0.62	1.42 ± 0.74	2.14 ± 0.79
Bupivacaine 0.25%	0.63 ± 0.9	1.13 ± 0.83	1.5 ± 1.5	1.9 ± 0.68
P-value	0.95	0.91	0.48	0.27

^a^ Values are expressed as mean ± SD.

As anticipated from the analysis, analgesic consumption post-surgery was higher in the bupivacaine 0.25% group compared to the bupivacaine 0.5% group (Table 3). The findings indicate that painkiller usage at 3 and 6 hours was lower in the bupivacaine 0.5% group; however, no significant difference was observed between the two groups (P = 0.052, χ^2^ = 3.77; P = 0.43, χ^2^ = 0.61; P = 0.31, χ^2^ = 1.02, respectively). However, this difference was significant at 12 hours post-surgery. The analgesic effect of bupivacaine diminished after 12 hours, leading to an increased need for supplemental pain relief among patients.

**Table 3.** Comparison of the Frequency of Receiving Analgesics After Surgery in Two Groups ^a^

Use of Painkiller	3 Hours	6 Hours	12 Hours	24 Hours
**Bupivacaine 0.5%**	4 (18.2)	17 (77.3)	9 (40.9)	1 (4.5)
**Bupivacaine 0.25%**	10 (45.5)	19 (86.4)	19 (86.4)	0 (0)
**P-value ** ^**b**^	0.052	0.43	0.002	0.91

^a^ Values are expressed as No. (%).

^b^ P-value is obtained from chi-square test or Fischer exact test.

## 5. Discussion

Using higher doses of bupivacaine is essential for reducing pain and narcotic consumption. However, multicenter studies with larger populations are needed. In our study, additional analgesic consumption 12 hours postoperatively was significantly higher in the bupivacaine 0.25% group compared to the bupivacaine 0.5% group. A multimodal analgesia approach to pain management is widely advocated by experts (15). Notably, the amount of opioid consumption immediately post-surgery is inversely correlated with the quality of the patient's recovery.

Although some studies have examined different doses of bupivacaine using various methods, research evaluating ACB with different concentrations of bupivacaine for post-TKA pain control remains limited. The optimal dose of bupivacaine for ACB is still unclear. In our study, pain levels between 6 and 24 hours postoperatively were lower in the 0.5% bupivacaine group compared to those who received 0.25%. In this regard, in a retrospective cohort study by Hagar et al. (16), different concentrations of local anesthetic were administered via peri-articular injection both alone and in combination with ACB. The investigation examined three groups: Peri-articular injection of 0.25% bupivacaine, peri-articular injection of 0.5% bupivacaine, and ACB combined with peri-articular injection of 0.25% bupivacaine. The findings showed that the total narcotic consumption was lower in the 0.5% peri-articular injection group compared to the other two groups.

Also, oral narcotics use was lower in the bupivacaine 0.5% group than in the bupivacaine 0.25% group. The number of analgesic prescriptions within six weeks post-surgery was also lower in the bupivacaine 0.5% group compared to the other two groups. However, no significant difference was observed in pain scores measured by the Visual Analog Scale (VAS) instrument. Opiate consumption decreased at a similar rate across all groups after reaching its peak (16). These results contrast with our study’s findings, which indicated a notable difference in pain management between the bupivacaine 0.25% and bupivacaine 0.5% groups. This discrepancy may be attributed to differences in the type of arthroplasty procedure or variations in patient characteristics.

However, Hagar et al.’s (16) findings indicated that the peak narcotic consumption was lower in the 0.5% bupivacaine group compared to the other two groups, potentially due to more effective pain control. Another study found that ACB with 0.25% bupivacaine effectively reduced postoperative pain and narcotics usage. Notably, single-shot ACB combined with periarticular infiltration accelerated post-TKA recovery and decreased narcotic consumption compared to local anesthesia (17). This suggests that lower doses of bupivacaine may still be effective in ACB. However, our study does not support this conclusion, as 0.5% bupivacaine was significantly more effective than 0.25% bupivacaine in reducing postoperative pain. In this regard, Kim et al.'s study showed that compared with FNB, a single-shot ACB with 15 mL of 0.5% bupivacaine and epinephrine 1/20000 improved pain scores and reduced morphine consumption (18).

Although limited research directly compares ACB using different doses of bupivacaine for postoperative pain control in TKA, several studies have investigated ACB in combination with periarticular injection for pain management. Grosso et al.'s study evaluated 155 TKA patients who received spinal anesthesia. Participants were divided into three groups: The ACB (15 mL of 0.5% bupivacaine), peri-articular injection (50 mL of 0.25% bupivacaine), and a combination of both methods. The findings revealed that patients who received ACB alone had significantly higher average pain scores and opioid consumption compared to the combined group (7).

It has been shown that the average pain score during the first 72 hours post-TKA was 3.24 in the liposomal bupivacaine group, compared to 3.83 in the ropivacaine group — a statistically significant difference. Additionally, at 36 hours post-surgery, mean pain scores were lower in the bupivacaine group than in the ropivacaine group (19). In this context, Lakra et al. compared post-TKA pain control using the ACB method with liposomal bupivacaine versus standard bupivacaine. Their findings indicated that medication usage and pain scores were significantly lower in the liposomal bupivacaine group on days 0, 1, and 2 after surgery (20).

Periarticular infiltration analgesic regimens that penetrate the anterior, medial, and posterior aspects of the knee provide pain relief for only 6 to 12 hours, with reinjections administered via intraoperative catheters (21). The limited duration of periarticular infiltrative procedures may be due to variability in blocking the posterior elements and distal geniculate nerves in the popliteal fossa (22). In a study by Nader et al., a significant number of patients in the active control group reported posterior knee pain as the site of perceived primary pain, possibly due to variability in the infiltration technique. Conversely, patients who received saline were more likely to experience pain in the anterior aspect of the knee, as the periarticular infiltration method in the aforementioned study mainly involved the posterior and medial capsule (17).

The findings of a study have shown that ACB does not cause adductor muscle weakness, whereas FNB is associated with a 49% reduction in quadriceps muscle tone in healthy volunteers (23). Additionally, Jæger et al. demonstrated that with the usage of ACB, no notable difference in quadriceps muscle strength was observed in people who received different volumes between 10 and 30 mL of 0.1% ropivacaine (23). We investigated two different doses of bupivacaine for ACB. The volume, concentration, and injection site of the anesthetic within the adductor canal can influence the spread of local anesthetic, its analgesic effect, and the potential for muscle weakness. A high volume or concentration of regional anesthetic may impact quadriceps muscle tone and possibly lead to sciatic nerve entrapment, while a low volume or concentration of local anesthetic in the adductor canal may not provide adequate pain relief (24).

Surgeons who favor peripheral anesthetic injection over ACB are concerned about surgical delay due to the use of ACB, increased costs, and minor risks associated with regional blocks. On the other hand, high-dose peri-articular anesthetic injection can increase the risks of systemic and cardiovascular toxicity. Given these factors, ACB remains a preferred choice among many surgeons.

Further research is needed to establish whether a significant correlation exists between the use of ACB and overall drug consumption following TKA. No cases of cardiovascular toxicity or dose-related adverse events related to systemic toxicity of local anesthetic were observed during the care period for the patients in this study, including those who received a 0.5% bupivacaine dose. This finding aligns with the results of Hagar et al.'s study (16). The study by Peterson et al. showed that a high dose equivalent to 60 cc of bupivacaine 0.5% did not cause any complications related to local anesthetic injection (25).

The retrospective cohort study by Melina Shon examined adult participants who underwent primary, unilateral TKA. Patients were divided into two groups: One group received a single-shot ACB alone (administered with 0.25% bupivacaine), while the second group received a combined single-shot ACB + IPACK (administered with 0.25% bupivacaine, 1 mg/kg dexmedetomidine, and 4 mg dexamethasone). Compared to ACB alone, patients who received combined single-shot ACB + IPACK had lower total narcotic consumption and reduced average pain scores during most of the immediate postoperative duration following primary, unilateral TKA (26).

Ilfeld et al. reported that 92% of trials suggested peripheral nerve block with unencapsulated bupivacaine provides superior analgesia to infiltrated liposomal bupivacaine (27). Similarly, findings from Hussain et al. indicated no significant differences between liposomal and plain bupivacaine LIA in extended post-surgery pain management, opioid use, or functional and safety outcomes on days 2 and 3 post-TKA. High-quality evidence does not support the use of liposomal bupivacaine for TKA (28). Overall, there is limited evidence of a toxic dose that leads to side effects when high-dose anesthetics are administered into the tissues around the joint.

This study designed by Kampitak et al. evaluated 140 patients undergoing TKA, comparing the effects of a 20 mL bolus of 0.25% bupivacaine versus a 10 mL bolus of 0.15% bupivacaine, both accompanied by continuous ACB and other analgesic methods. The primary outcome measured knee pain at 6 and 12 hours postoperatively, with a non-inferiority margin of 1 point. Results showed no significant differences between the groups. Secondary measures — including rest/movement pain, morphine use, and time to first rescue analgesia — also revealed no clinical differences (29).

The findings suggest that while a lower-dose regimen (10 mL of 0.15% bupivacaine) provides comparable pain relief with potential medication reduction, our results showing notably higher opioid consumption in the 0.25% group at 12 hours post-surgery (P = 0.002) indicate the lower-dose group may have required additional analgesia. This raises the possibility that higher concentrations offer better extended pain control, though outcomes may vary by study design and patient factors.

Our findings contrast with another trial evaluating 133 mg vs. 266 mg liposomal bupivacaine, which found a significant reduction in NRS scores at rest but no difference in pain levels during activity. While sensory and motor block onset times were notably shorter with the higher dose, opioid consumption differences were confined to the early postoperative period, with no significant variance in overall usage (30).

Taken together, these results suggest that higher doses of bupivacaine may offer superior prolonged pain relief, particularly at later time points, without negatively impacting rehabilitation quality or satisfaction. However, since similar opioid use beyond the initial postoperative phase, optimizing bupivacaine concentration should be weighed against potential side effects, economic considerations, and individualized patient needs. Future studies with larger sample sizes and longer follow-up periods will be valuable in refining optimal dosing strategies for TKA pain management.

While our study provides valuable insights into the effectiveness of ACB with different bupivacaine concentrations for post-TKA analgesia, several limitations should be acknowledged. First, the sample size was relatively small, with only 44 participants, which may limit the generalizability of the findings. A larger, multicenter trial would strengthen the reliability of the results. Second, while VAS pain scores and opioid consumption were measured, additional functional assessments, such as gait analysis and long-term rehabilitation outcomes, were not included, which would provide a more comprehensive understanding of the impact of different bupivacaine concentrations on recovery. Finally, although opioid consumption was recorded, the study did not account for other adjunct analgesic methods that patients might have used, which could influence postoperative pain scores. Future studies with larger populations, extended follow-up periods, additional functional assessments, and stricter blinding protocols are necessary to further validate and optimize ACB dosing strategies in TKA recovery.

### 5.1. Conclusions

Our study demonstrates that while pain intensity at 3 hours post-surgery was comparable between groups, the 0.5% bupivacaine group exhibited significantly lower VAS scores at later time points (6, 12, and 24 hours) compared to the 0.25% bupivacaine group (P = 0.02, P < 0.005, P = 0.002, respectively). Despite this improved pain control, patient satisfaction and quadriceps muscle strength did not differ significantly, suggesting that the enhanced analgesia did not directly impact functional recovery or subjective experience.

## Data Availability

The dataset presented in the study is available on request from the corresponding author during submission or after publication.

## Clinical Trial Registration Code:

IRCT20220731055587N1.
